# Low Immunogenicity of Neural Progenitor Cells Differentiated from Induced Pluripotent Stem Cells Derived from Less Immunogenic Somatic Cells

**DOI:** 10.1371/journal.pone.0069617

**Published:** 2013-07-26

**Authors:** Pengfei Liu, Shubin Chen, Xiang Li, Li Qin, Ke Huang, Lihui Wang, Wenhao Huang, Shengbiao Li, Bei Jia, Mei Zhong, Guangjin Pan, Jinglei Cai, Duanqing Pei

**Affiliations:** 1 Key Laboratory of Regenerative Biology, Guangdong Provincial Key Laboratory of Stem Cell and Regenerative Medicine,Guangzhou Institute of Biomedicine and Health, Chinese Academy of Sciences, Guangzhou, Guangdong Province, P.R. China; 2 Department of Regeneration Medicine, School of Pharmaceutical, Jilin University, Changchun, Jilin Province, P.R. China; 3 Laboratory of Pathogen Biology, State Key Laboratory of Respiratory Disease, Center for Infection and Immunity, Guangzhou Institutes of Biomedicine and Health, Chinese Academy of Sciences, Guangzhou, Guangdong Province, P.R. China; 4 The Center for Prenatal and Hereditary Disease Diagnosis, Department of Obstetrics and Gynecology, Nanfang Hospital, Southern Medical University, Guangzhou, Guangdong Province, China; University of Pécs Medical School, Hungary

## Abstract

The groundbreaking discovery of induced pluripotent stem cells (iPS cells) provides a new source for cell therapy. However, whether the iPS derived functional lineages from different cell origins have different immunogenicity remains unknown. It had been known that the cells isolated from extra-embryonic tissues, such as umbilical cord mesenchymal cells (UMCs), are less immunogenic than other adult lineages such as skin fibroblasts (SFs). In this report, we differentiated iPS cells from human UMCs and SFs into neural progenitor cells (NPCs) and analyzed their immunogenicity. Through co-culture with allologous peripheral blood mononuclear cells (PBMCs), we showed that UMCs were indeed less immunogenic than skin cells to simulate proliferation of PBMCs. Surprisingly, we found that the NPCs differentiated from UMC-iPS cells retained low immunogenicity as the parental UMCs based on the PBMC proliferation assay. In cytotoxic expression assay, reactions in most kinds of immune effector cells showed more perforin and granzyme B expression with SF-NPCs stimulation than that with UMC-NPCs stimulation in PBMC co-culture system, in T cell co-culture system as well. Furthermore, through whole genome expression microarray analysis, we showed that over 70 immune genes, including all members of HLA-I, were expressed at lower levels in NPCs derived from UMC-iPS cells than that from SF-iPS cells. Our results demonstrated a phenomenon that the low immunogenicity of the less immunogenic cells could be retained after cell reprogramming and further differentiation, thus provide a new concept to generate functional lineages with lower immunogenicity for regenerative medicine.

## Introduction

The successful establishment of human embryonic stem cells (hES cells) proved a decisive turning point in biomedical science, providing a renewable source of various cell types for human cell therapy [1]. hES cells derived from early blastocysts are pluripotent and able to differentiate into all cell types present in the body [1], [2]. The differentiated products of hES cells have been used successfully in animal models of diseases, injury and aging, such as myocardial infarction [3], ischemic-reperfusion injury [4], Parkinson's disease [5], [6], spinal cord injury [7], and macular degeneration [8], [9]. While highly promising, several challenges have been raised in hES-based therapy, such as the ethical issue, low efficacy in establishment, and immune rejection with allogeneic transplantation. These challenges are overcome by the recent breakthrough of induced pluripotent stem cells (iPS cells) reprogrammed from somatic cells with defined factors (Oct4, Sox2, Klf4, and c-Myc) [10]. The iPS cells with unlimited growth capacity have similar characteristics to ES cells, such as multi-lineage *in vitro* differentiation, teratoma formation, germline transmission, and even contribution to entire animals [11]–[14]. With the development of iPS techniques, the somatic cells from different species and various tissues were reprogrammed successfully [15]–[20]. Importantly, the autologous cells derived from one's own iPS cells are theoretically immune tolerant, and have opened new avenues in autologous cell and tissue transplantation [21]–[23]. Therefore, iPS cells opened new opportunities in biomedical research.

When it comes to studying and treating human diseases, iPS cells are considered potentially far more useful than ES cells. It is widely believed that they could be generated by taking cells from a patient, treating them, and inducing them into therapeutic cells that can be returned to the same individual without the risk of rejection [21]–[23]. For examples, researchers have already taken the iPS cells created from patients with neurodegenerative diseases and beta-thalassemia and converted them into neurons [24], [25] and hematopoietic progenitors [26]. Moreover, researchers have taken the next step, the neural cells and the genetically corrected iPS-derived hematopoietic progenitors were used in animal models of sickle-cell anaemia, Parkinson's disease [24], [27]and sub-lethally irradiated immune deficient SCID mice, respectively [26]. However, Dr. Fairchild has expressed concerns about the potential immunogenicity of iPS and its derived cell types as early as 2010 [28]. In 2011, Zhao et al. reported that the transplantation of undifferentiated iPS cells induced a T-cell-dependent immune response even in a syngeneic mouse [13]. The authors also revealed several genes, such as Zg16 and Hormad1, directly contributed to the immunogenicity of iPS derivatives in its syngeneic mouse in the T-cell-dependent immune manner. However, undifferentiated iPS cells, which can randomly differentiate into teratomas, likely cannot be used for medical applications. Thus, it may not be surprising that there are T-cell infiltration in the developing teratomas [29]. Nevertheless, it is entirely possible that this immunogenicity could further increase during differentiation to specific tissues, as has been observed during differentiation of ES cells with increasing expression of HLA [30]–[33]. A recent study has demonstrated that upregulated expression of NFκB1 and RelA, two members of NFκB family during cell reprogramming, could increase the expression of HLA-I in iPS cells [34]. Suárez -Alvarez et al. have shown that revealed HLA-B and β-2M can activate the transporter associated with antigen processing and can thus increase immunogenicity through induction of H3K4me3 modification during the differentiation [35]. Recently, Araki et al. showed limited or no immune responses, including T-cell infiltration, for tissues derived from either iPS or ES cells in the hosts [36]. Moreover, no increase was observed in the expression of the immunogenicity-causing Zg16 and Hormad1 genes in regressing skin and bone marrow tissues, either [36]. However, whether autologous human iPS-derived differentiated cells have no immune responses has not yet been strictly examined. It is suggested that the immunogenicity of the human iPS-derived terminally differentiated cells could be tested by transplantation into the gene matching mice with a human immune system [37].

From a practical point of view, the autologous derivation of iPS cells would require a lot of time and cost for assessment of their medical stability, safety, and efficacy [30], [34], [38]. Thus, generation of universal donor cells was raised as another hope for regenerative medicine [34], [38], [39]. For example, the umbilical cord is extra-embryonic tissue of particular interest for regenerative medicine [12], [15]. It can serve as a source of large numbers of cells with mutilineage differentiation potential that are poorly immunogenic and without controversy [40]–[42]. Moreover, when compared with juvenile or adult donor cells such as spleen fibroblasts, umbilical cord mesenchymal cells (UMCs) likely carry much fewer mutations, including silent and frameshift mutations [15], [43]. Previously, we have succeeded in reprogramming human UMCs into iPS cells (UMC-iPS cells), which was found to share a highly similar transcriptome profile with H9-hES cell line by DNA arrays [15]. However, the immunogenicity of UMC-iPS cells and its derivatives remains unclear.

In our study, we hypothesized UMC-iPS derivatives had lower immunogenicity than those from other origins, such as adult skin fibroblasts (SFs). Thus, we here compared the immunogenicity of three cell stages from both SFs and UMCs as follows, somatic cells (SFs and UMCs), somatic cell derived iPS cells (SF-iPS cells and UMC-iPS cells), and iPS derived NPCs (SF-NPCs and UMC-NPCs). Our data demonstrated that iPS cells derived from less immunogenic starting cells, such as UMCs, result in less immunogenic derivatives after reprogramming and after further neural differentiation.

## Results

### Neural differentiation from human ES/iPS cells

We induced human ES/iPS cells for neural differentiation as described [15], [44], [45]. Typical neural rosettes were generated in the 3rd week, followed by formation of neural spheres consisting of neural progenitor cells (NPCs) (Figure S1A, S1C). These proliferating hES/iPS-derived NPCs were purified by passaging every 5 days. Both SF-iPS and UMC-iPS derived NPCs (SF-NPC, UMC-NPC) expressed similarly high levels of the NPC marker Nestin as that detected in HN4-hES derived NPCs (Figure S1B). Another NPC marker Forse1 also showed high expression level (>70% positivity) in both ES and iPS derived NPCs (Figure S1B). Moreover, these NPCs could be further differentiated into neuronal-like and astrocyte-like cells, being stained positive for neuronal (βIII-tubulin) and astrocyte markers (GFAP) respectively (Figure S1C). Therefore, our hES/iPS derived NPCs using in the following investigation not only display adequate NPC imprinting, but also have the ability to differentiate into neural lineage cells.

### Proliferation of PBMCs in co-culture system

**Figure 1:**
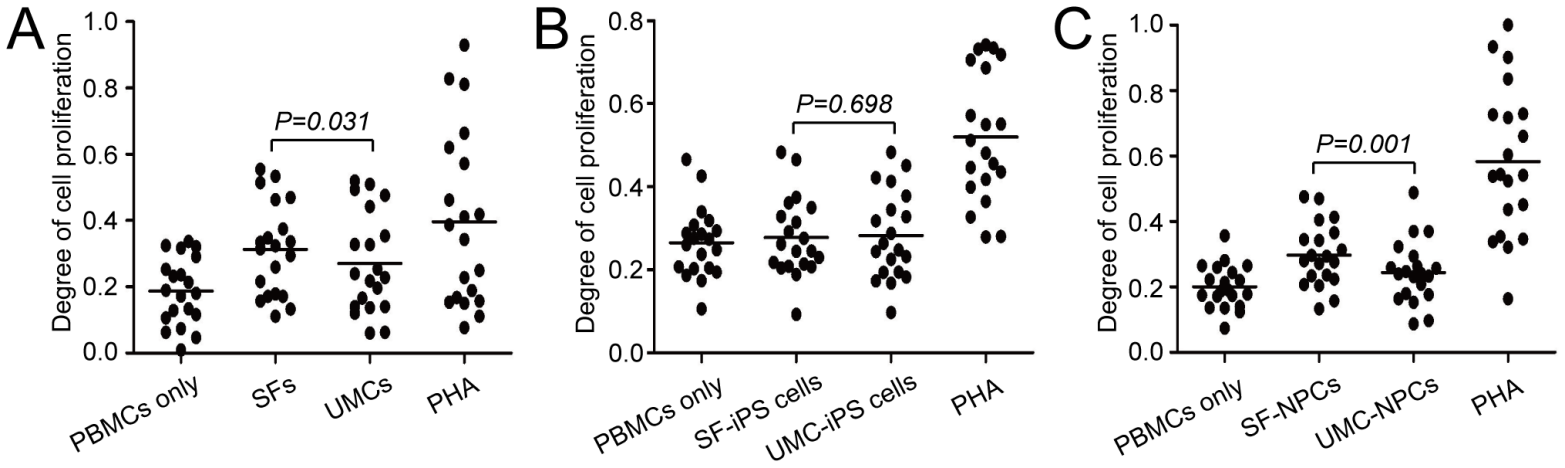
Proliferation of PBMCs stimulated by different cell types derived from SF and UMC in co-culture system. (A) PBMC proliferation stimulated by different parental somatic cells (SFs and UMCs). (B) Proliferation of PBMCs stimulated by SF-iPS and UMC-iPS cells. (C) Proliferation of PBMCs stimulated by SF-NPCs and UMC-NPCs. PBMCs randomly from 20 healthy volunteers from a 20–40 years old population were used for each cell type couple. Negative control group contained PBMCs only and positive control group contained PBMCs supplemented with PHA. Differences between groups were analyzed by paired-samples *t*-tests with statistical significance set at 0.05.

To determine whether the low immunogenicity of UMCs could be retained after cell reprogramming and further differentiation, we used a co-culture system of target cells and PBMCs to investigate PBMC proliferation in SF-group (SFs, SF-iPS, SF-NPCs) and UMC-group (UMCs, UMC-iPS, UMC-NPCs). For identification of the suitable proportion between target cells and PBMCs in co-culture system, each somatic cell (SF or UMC) was co-cultured with PBMCs obtained from 5 volunteers, respectively. Allogeneic cell proliferation with glomerulus-like cell masses was observed when somatic cells were incubated with PBMCs at the ratios of 1∶1, 1∶5, 1∶10, 1:25 and 1∶50. PBMC proliferation was present in the co-culture system at the ratio of 1∶100. We further found that the ratio of 1∶100 showed similar tendency to that of 1∶1000 between UMCs and SFs in the PBMC culture system (Figure S2). Thus, we performed target cell/PBMCs co-culture at the ratio of 1∶1000. PBMCs from 20 healthy volunteers were isolated to co-culture with various cell types for 3 days as follows, parental somatic cells (SFs and UMCs), iPS cells (SF-iPS and UMC-iPS cells) and iPS-derived NPCs (SF-NPCs and UMC-NPCs). As a positive control, we observed that PBMCs proliferation was highly increased by PHA simulation, and it showed the significant difference in proliferation from the negative control group. For somatic cells, suppression of PBMCs proliferation was present in UMC stimulated group. The proliferation of PBMCs stimulated by SFs was higher than that stimulated by UMC (average degree of cell proliferation: 0.313 vs. 0.271, *P = 0.031*, Figure 1A, Table S1). A similar result was detected in NPC groups for comparing SF-NPCs to UMC-NPCs (average degree of cell proliferation: 0.297 vs. 0.244, *P = 0.001*, Figure 1C, Table S1). These findings showed that UMCs and UMC-NPCs have lower immunogenicity than SFs and SF-NPCs, respectively. These data showed that lower immunogenicity was retained in the differentiated cells derived from the less immunogenic somatic cells through iPS generation and further differentiation. On the other hand, when PBMCs were co-cultured with SF-iPS or UMC-iPS cells, no significant difference in incorporation of BrDU was observed, but closed degree of cell proliferation were achieved (average degree of cell proliferation: 0.278 vs. 0.282, *P = 0.703*, Figure 1B, Table S1). In addition, proliferation of iPS cell groups showed a level similar to that observed in the negative control group (average degree of cell proliferation: 0.265). No obvious differences existed between negative control group and two iPS cell groups respectively (compare to SF-iPS group, *P = 0.203*; compare to UMC-iPS group, *P = 0.133*, Figure 1B). These data indicated iPS cells from whatever origins should be less immunogenic.

### Distinct expression kinetics of perforin and granzyme B in co-culture system

**Figure 2:**
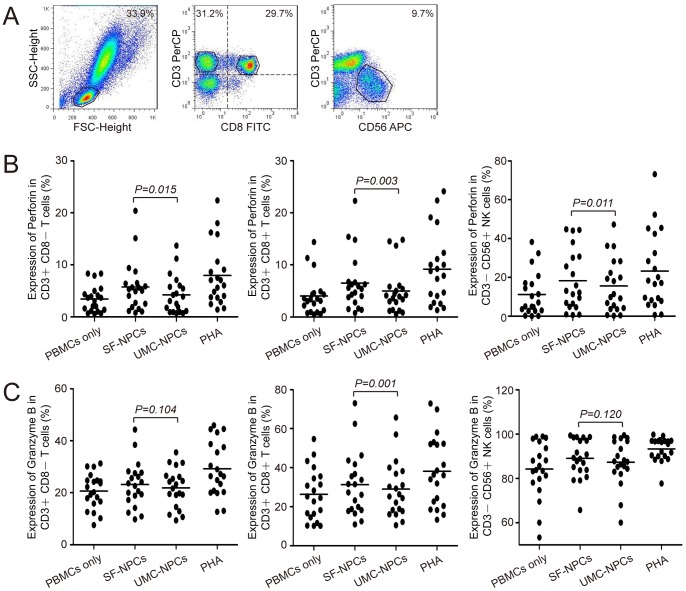
Perforin and granzyme B expressions in PBMCs co-culture system with NPC stimulation. (A) Analysis of surface phenotypic markers (CD3, CD8, CD56) of T cells and NK cells. PBMCs (n = 20) co-cultured with SF-NPC or UMC-NPC were labeled with different fluorescence-conjugated antibodies, dividing into three obvious cell groups (CD3+CD8− T cells, CD3+CD8+ T cells and CD3−CD56+ NK cells) monitored by flow cytometry analysis. (B and C) Analysis of perforin and granzyme B expression in T cells and NK cells. Differences between groups were analyzed by paired-samples *t*-tests (*P*<*0.05*).

To further determine whether the distinct differences in the kinetics of perforin and granzyme B happened in co-culture system, we compared the expression of perforin and granzyme B in PBMCs and T lymphocytes stimulated with iPS derived NPCs (SF-NPCs and UMC-NPCs). The percentage of perforin and granzyme B expression in CD3+CD8− T cells, CD3+CD8+ T cells and CD3−CD56+ NK cells were analyzed in co-cultured PBMCs or T lymphocytes by flow cytometry (Figure 2A, 3A). Distinguishable differences of both perforin and granzyme B expressions were observed between positive and negative control groups.

**Figure 3:**
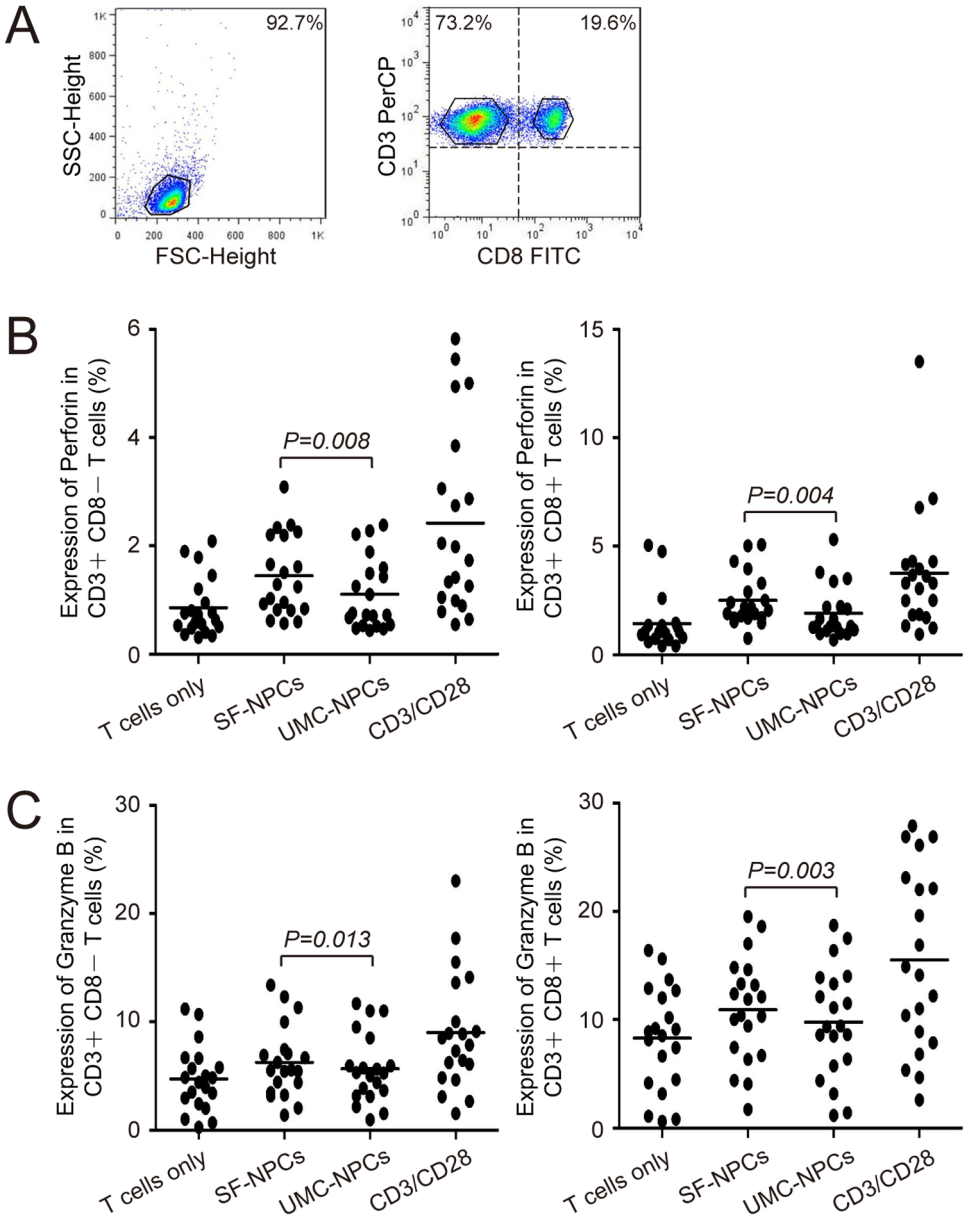
Perforin and granzyme B expressions in T lymphocytes co-culture system with NPC stimulation. (A) Analysis of surface phenotypic markers (CD3, CD8) of T cells. T lymphocytes (n = 20) co-cultured with SF-NPC or UMC-NPC were labeled with different fluorescence-conjugated antibodies, dividing into two obvious cell groups (CD3+CD8− T cells and CD3+CD8+ T cells) monitored by flow cytometry analysis. (B and C) Analysis of perforin and granzyme B expression in T cells. Differences between groups were analyzed by paired-samples *t*-tests (*P*<*0.05*).

In the case of PBMCs co-culture system, more perforin was expressed with SF-NPCs stimulation than that with UMC-NPCs stimulation in all three cell groups (CD3+CD8- T cells, CD3+CD8+ T cells and CD3−CD56+ NK cells). The average fluorescence intensity of perforin-expressing cells showed significant differences between the groups stimulated by SF-NPCs and UMC-NPCs (CD3+CD8− T cells: 5.72% vs. 4.22%, *P = 0.015*; CD3+CD8+ T cells: 6.52% vs. 4.98%, *P = 0.003*; CD3−CD56+ NK cells: 18.30% vs. 15.54%, *P = 0.011*, Figure 2B, Table S2). In addition, the expression level of granzyme B in CD3+CD8+ T cells was decreased in UMC-NPCs stimulated group when compared to that of SF-NPCs stimulated group (31.39% vs. 28.96%, *P = 0.001*, Figure 2C, Table S3). However, no distinct difference in granzyme B expression was present between two NPCs-stimulated groups on the other two kinds of immune cells (CD3+CD8− T cells: 23.11% vs. 21.89%, *P = 0.104*; CD3−CD56+ NK cells: 89.01% vs. 87.33%, *P = 0.120*, Figure 2C, Table S3). The results suggested that SF-NPCs are able to activate immune cells and induce immune response more easily than UMC-NPCs.

For direct T cell response assay, we isolated CD3+ cells from PBMCs and co-cultured these cells with both iPS derived NPCs, respectively. Similar to PBMCs co-culture system, the expression of perforin in T lymphocytes was significantly different between the groups stimulated by SF-NPCs and UMC-NPCs (CD3+CD8− T cells: 1.45% vs. 1.11%, *P = 0.008*; CD3+CD8+ T cells: 2.51% vs. 1.90%, *P = 0.004*, Figure 3B, Table S4). A distinct difference in granzyme B expression was observed between two NPCs-stimulated groups in both kinds of T cells (CD3+CD8− T cells: 6.27% vs. 5.67%, *P = 0.013*; CD3+CD8+ T cells: 10.91% vs. 9.76%, *P = 0.003*, Figure 3C, Table S5). Taken together, lower expression level of perforin and granzyme B existed in T lymphocytes compared with that in PBMCs.

### The expression level of multiple immunological factors showed distinct difference between SF-NPCs and UMC-NPCs

**Figure 4:**
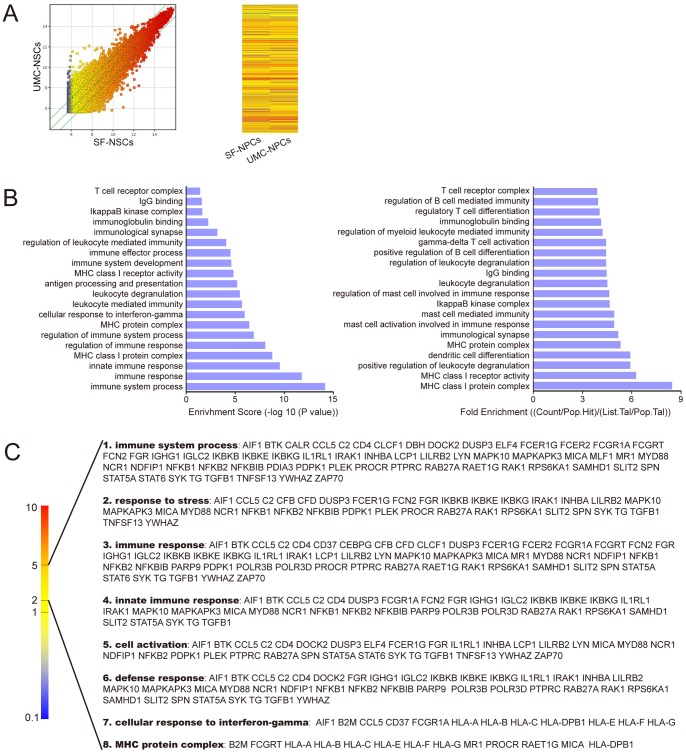
RNA microarray analysis of SF-NPCs and UMC-NPCs. (A) Comparison of global gene expression profiles of a SF-NPC line and a UMC-NPC line. The values of X and Y axes in the Scatter-Plot were normalized signal values of each sample (log2 scaled). The green lines are Fold Change Lines (The default fold change value given was 2.0). Over two fold alterations of genes between two compared samples could be found above the top green line and below the bottom green line. In Hierarchical Clustering for “all targets value”, “Red” indicates high relative expression, and “blue” indicates low relative expression. (B) Analysis of Gene Ontology project associated with immune process and relative molecular function. The bar plots showed the top twenty Fold Enrichment value and Enrichment Score value of the significant enrichment terms. (C) Summary of genes associated with immune system and gene ontology terms. Strip from “Red” to “Blue” indicates the fold of immune genes expressed in SF-NPC compared to UMC-NPC. The fold of gene expression between two compared samples mainly varied between 2 and 5, showing the relation with immune system process, response to stress, immune response and so on.

Gene expression in both SF-NPCs and UMC-NPCs was verified by RNA arrays (Figure 4). The Scatter-Plot and Hierarchical Clustering were performed based on all target values to assess the difference in gene expression between the SF-NPCs and UMC-NPCs, and the results showed distinguishable gene expression profiling between the two samples (Figure 4A). The Gene Ontology project provides a controlled vocabulary to describe genes, associated with immune process and immune molecular function, up regulated in SF-NPCs compared to UMC-NPCs. The bar plots showed the top twenty Fold Enrichment value and Enrichment Score value of the significant enrichment terms (Figure 4B). Over 70 genes associated with immune system showed about 2–5 times higher expression in SF-NPCs than those in UMC-NPCs and the relevant immunological function was summarized (Figure 4C). The results showed differences in gene expression between SF-NPCs and UMC-NPCs, including expression of HLA and factors in NFκB family.

### Expression of HLA in human somatic cells, iPS cells, and NPCs differentiated from iPS cells

**Figure 5:**
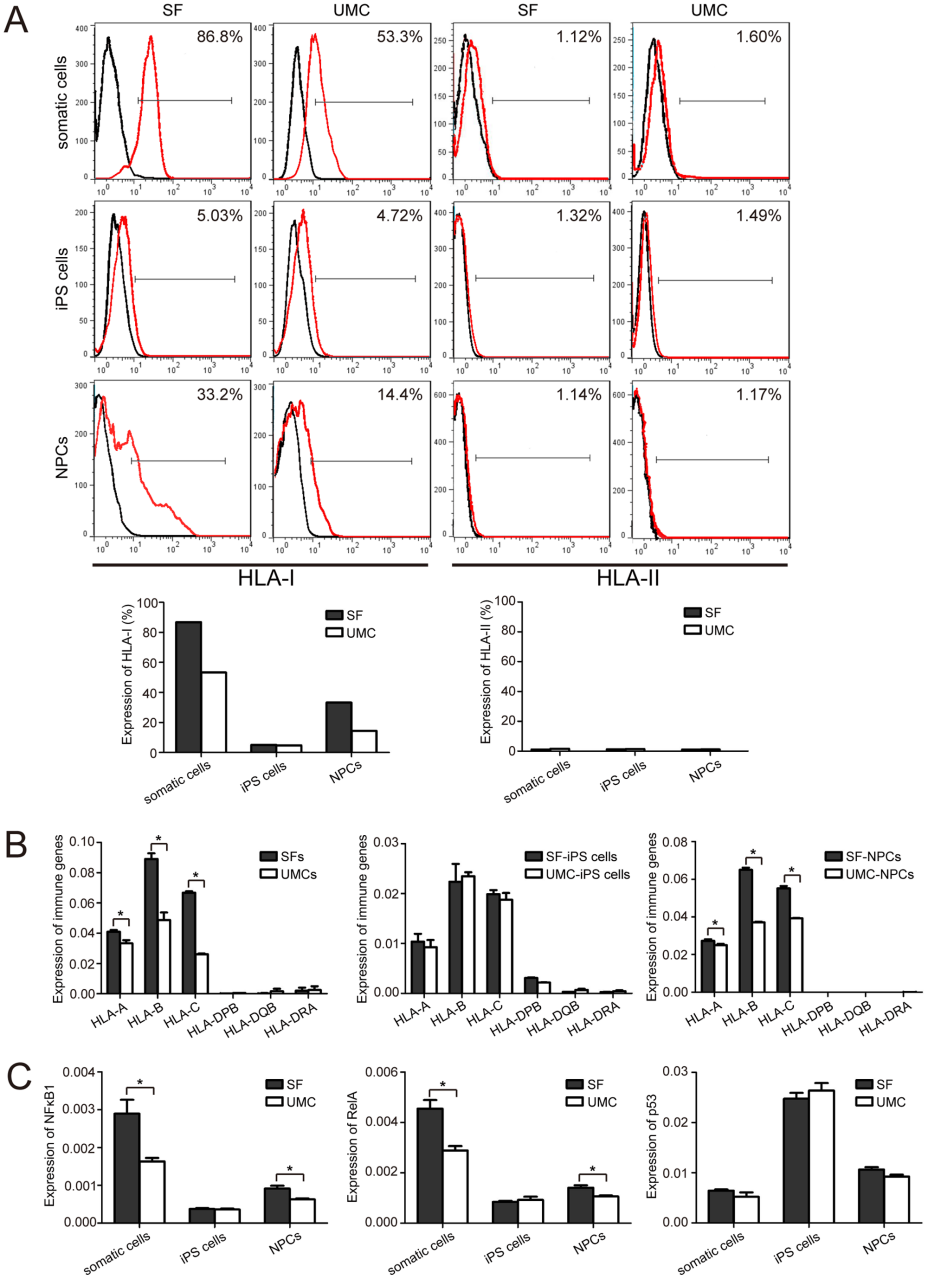
Expressions of HLA-I/II and nuclear factors in somatic cells, iPS cells and NPCs derived from SF and UMC origins. (A) Flow cytometry analysis of HLA-I/II expression in different cell types derived from SFs and UMCs. Top: SF and UMC somatic cells; middle: SF-iPS and UMC-iPS cells; bottom: SF-NPCs and UMC-NPCs. Expression variation of HLA-I/II at three stages of somatic cells, iPS cells, and NPCs was analyzed in the histograms based on the data of flow cytometry analysis. (B) qPCR detection of HLA-I/II in three different cell types of SFs and UMCs, matching the tendency of flow cytometry analysis. (C) Expression of NFκB1, RelA and p53 in different cell types derived from SFs and UMCs. Similar qPCR results were obtained in at least three independent experiments. Results were expressed as mean ± SEM. A *t*-test was used to compare the various groups and *p*-value less than 0.05 were considered statistically significant. * *P*<*0.05*.

Based on the data obtained in RNA array, we further investigated the expression of HLA-I and -II in the two cell origins (SFs and UMCs) at their three stages (somatic cells, iPS cells, iPS-derived NPCs). In the case of smatic cells, reaction with HLA-I antibody showed lower levels of HLA-I expression in UMC (Figure 5A). The expression levels of HLA-I protein in both SF-iPS and UMC-iPS cells were detected at a level as low as that of human ES cell line (Figure 5A). We then examined whether the iPS derived NPCs would lead to the up-regulation of HLA-I. As we expected, NPCs derived from both SFs and UMCs resulted in an elevation of HLA-I expression. In particular, expression of HLA-I protein in UMC-NPCs was still obviously lower than that in SF-NPCs (Figure 5A). The data of flow cytometry were further analyzed in a bar graph. We could find easily that SFs expressed higher levels of HLA-I than UMCs did, which were found to appear in SF- and UMC-NPCs as well; while both iPS cell lines expressed significantly lower levels of HLA-I than their parental somatic cells and derived NPCs (Figure 5A). On the other hand, much lower expression of HLA-II was observed in somatic cells, iPS cells and NPCs from both cell sources (Figure 5A). In addition, the expression of HLA-I and -II at RNA level was verified via qPCR (Figure 5B). The data were similar to those obtained by flow cytometry. Expression of HLA-A/B/C in both SFs and SF-NPCs was higher than that in UMCs and UMC-NPCs, respectively (*P<0.05*). However, no obvious differences in the expression of HLA-A/B/C could be detected between SF-iPS and UMC-iPS cells. As expected, HLA-DPB/DQB/DRA expression kept a much lower level in all groups.

To further analyze effects of cytokine on HLA-I and -II expression of different NPCs, we added IFN-γ in NPCs/PBMCs co-culture system, which mimicked a similar environment of inflammation or damaged tissues after transplantation *in vivo*. After addition of 25 ng/ml IFN-γ for 48 h, it resulted in a dramatic up-regulation in the expression of HLA-A/B/C and HLA-DPB/DQB/DRA in SF-NPCs and UMC-NPCs. The expression of HLA-A/B/C and HLA-DPB/DQB in PBMCs stimulated by SF-NPCs was higher than that stimulated by UMC-NPCs (Figure S3). Taken together, the expression of HLA-I is suppressed during the reprogramming and is activated during the differentiation.

As reported, HLA-I could be regulated by NFκB1 and RelA. We then investigated whether the expressions of NFκB1 and RelA have similar tendency as that of HLA-I between SFs and UMCs at their stages of somatic cells, iPS cells and iPS derived NPCs. Our data showed indeed to match nearly the variation tendency of HLA-I caused by cell reprogramming and further differentiation. Compared with UMC derived cells, the higher expression of both NFκB1 and RelA could be detected in both somatic cells and NPCs derived from SF (Figure 5C). In addition, p53 was correlated with the ability of NFκB family, and this gene usually holds a higher expression in the cells with a lower expression of NFκB family [46], [47]. In our results, the expression level of p53 was highest in iPS cells, then in NPCs, but lower in somatic cells. However, no significant difference exited between SFs and UMCs, SF-iPS cells and UMC-iPS cells, or SF-NPCs and UMC-NPCs (Figure 5C). This indicated that the NFκB family regulated the variation of cell immunogenicity, while the function of p53 here remained unclear. We further examined the expression of Zg16 and Hormad1, which were not detected to express in somatic cells, iPS cells and derived NPCs in both SF- and UMC- groups (data not shown). This indicates the iPS derived NPCs in our model may not have distinguished abnormal expression of immunogenic proteins. Taken together, it is suggested that the regulation of NFκB be involved in the determination of immunogenicity in reprogramming and differentiation.

## Discussion

**Figure 6:**
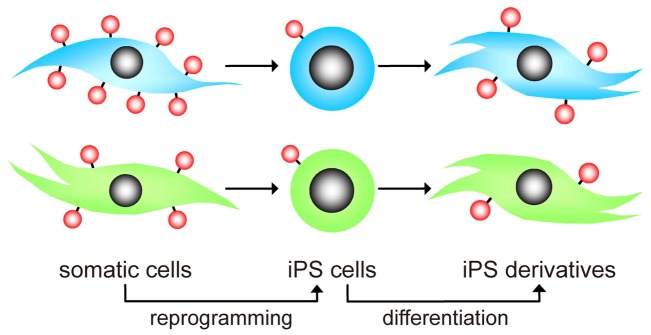
Schematic representation of variation in immunogenicity detected in somatic cells after iPS generation and further differentiation. The red dots represent HLA-I expressed in somatic cells, their iPS cells and the iPS derivatives. Expression of HLA-I was decreased by the reprogramming event and increased during differentiation. Different somatic cells may have different immunogenicity (high or low), displaying as HLA-I expression. After reprogramming into iPS cells, the HLA-I expression reduces into a lower level regardless of cell origins. Different expression levels of HLA-I appear again when these iPS cells differentiate. iPS cells derived from less immunogenic somatic cells result in less immunogenic iPS derivatives, and vice versa. Blue cells represent more immunogenic cell types; green cells represent less immunogenic cell types.

Here we describe that the less immunogenic somatic cells retain their low immunogenicity in their iPS derivatives. With the diversity of starting cells (somatic cells) available to produce iPS cells, the less immunogenic starting cells derived iPS cells could lead to the less immunogenic differentiated cells (Figure 6).

PBMCs include T cells, B cells, NK cells, monocytes/macrophages and dendritic cells, which perform a range of different effector functions during the immune rejection [48], [49]. We examined the proliferation of this mixed cell population when stimulated by different cell stages from SFs and UMCs respectively. Our data confirmed that suppression of PBMCs were present in the group stimulated by the UMC-NPCs derived from the less immunogenic starting cells UMCs, when compared with the group stimulated by SF-NPCs. This tendency was also detected in the somatic cell groups. Besides PBMCs proliferation, we observed a lower release of cytotoxic molecules such as perforin and granzyme B occurred in the less immunogenic cell types. As expected, our data showed less perforin expression of UMC-NPCs stimulated group in all three cell groups (CD3+CD8− T cells, CD3+CD8+ T cells and CD3−CD56+ NK cells), while the effective difference of granzym B expression was only detected in CD3+CD8+ cells in PBMCs co-culture system. These results indicate that UMC-NPC is less immunogenic than SF-NPC.

Not only cell proliferation but also the expression of cytotoxic molecules in PBMCs should have been caused by cross-presentation of NPC-derived allogeneic antigens by antigen presenting cells included in the PBMCs. In fact, both direct T cell response and indirect T cell responses are critically involved in graft rejection in the transplantation immunity. Regarding the T cell co-culture assay, expression of perforin and granzyme B in both CD3+CD8− T cells and CD3+CD8+ T cells kept similar tendency as that in PBMC co-culture system. However, lower expression level of cytotoxic molecules existed in T lymphocytes than that in PBMCs. Without cross-presentation by antigen presenting cells, direct T cell response was lower than the indirect T cell response. From another point of view, the expression of cytotoxic molecules between CD3+CD8− and CD3+CD8+ T cells displayed different results in different antigen stimulated groups. Equivalent cytotoxic potential based on perforin expression was detected between the above two kinds of T cells in both negative control and UMC-NPC stimulated groups of PBMC co-culture system (* and # in Table S2). Nevertheless, higher expression levels of cytotoxic molecules were observed in CD3+CD8+ T cells in both the PBMC and T cell co-culture systems (Table S2–S5). It is known that CD3+CD8− T cells contain CD4+ T cells and NKT cells. Although there are only few reports that CD3+CD8− T cells possess cytotoxic potential, recent studies have demonstrated that CD4+ T cells can also exhibit a strong cytotoxic activity that results in inhibition of tumor development [50]–[55]. Thus, these data suggest that under certain conditions CD3+CD8− T cells may have cytotoxic potential similar to that observed for CD3+CD8+ cytotoxic T cells.

Furthermore, microarray analysis showed higher expression level of immune genes in SF-NPCs compared with UMC-NPCs. These genes were associated with immunological processes and factors, including HLA, NFκB, MAPK, and TGF-β. In particular, all members of HLA-I are included, showing higher expression level in SF-NPCs. The HLA-I expression of both UMC-iPS and SF-iPS cells showed a similar expression level, which is lower than that of somatic cells. When differentiated into NPCs, the HLA-I expression was reactivated, while UMC-NPCs expressed a lower level of the HLA-I complex than SF-NPCs. Our findings are consistent with data from earlier reports that described lower PBMC proliferation in the presence of UMCs, when compared to the negative control group [41], [42]. These data also indicate that the final immunogenicity of iPS-derived cells is, at least partly, determined by the level of immunogenicity of the parental somatic cells.

Moreover, it remains to be determined whether abnormal expression of antigens, including HLA, may accompany the reprogramming processes during generation of iPS cells and their subsequent differentiation. Recent research indicated a significant positive correlation between MHC-I and the nuclear factors, NFκB1 and RelA, pointing to the critical role of NFκB proteins in regulating the MHC-I expression in human iPS cells [34]. In their study, Pick and colleagues reported that over-expression of NFκB1 and RelA in undifferentiated iPS cells could lead to induction of MHC-I expression and silencing of NFκB1 and RelA by shRNA results in decreased expression of β-2M after IFN-γ treatment. In our study, the expression levels of NFκB1 and RelA matched the variation tendency of HLA-I expression at three stages (somatic cells, derived iPS cells, and iPS derived NPCs) of derived from UMCs and SFs. Briefly, both NFκB1 and RelA showed lowest expression level in iPS cells, compared to somatic cells and NPCs. Particularly, the expression levels of NFκB1 and RelA in UMCs and SFs correlated with the levels in of these factors in the respective cell population derived from these parental cell types. Our data suggest that the different expression levels of NFκB1 and RelA between the UMC and SF somatic cells can be retained during the reprogramming and subsequent differentiation events. Since NFκB proteins have been reported to regulate MHC-I expression, it suggests that the level of immunogenicity could be retained to some degree during reprogramming and cell differentiation.

In addition, UMCs studied herein were reported to be able to differentiate into functional hepatocyte-like cells with retaining their low immunogenicity *in vitro*, which is consistent with our findings [12]. However, the underlying mechanism had not been explained. If the phenomenon of retention of low immunogenicity during cell reprogramming and differentiation could be considered as a kind of “memory”, what could underlie the regulation this type of memory? Epigenetic memory of the tissue origin is likely to affect the efforts of using iPS cells in various applications [56]–[58]. For example, iPS cells derived from adult murine tissues harbour residual DNA methylation signatures characteristic of their somatic tissue of origin, which favours their differentiation along lineages related to the donor cell, while restricting alternative cell fates. However, the epigenetic memory of immunogenicity remains to be fully elucidated. Thus, the phenomenon described in our report warrants further studies.

For stem cell therapy, the ability to generate the required cell type for each patient is still far from reality because of the length of time needed for the cell reprogramming, cost, and because of inherent difficulty in generating normal iPS cells from patients affected by genetic diseases [28], [39]. Some scientists suggested that the most suitable option for using iPS cell in transplantation is turning to cell banking [28], [39], [59]. Boyd and Wood have suggested that the less immunogenic starting cells should be used for allo-transplantation [60]. We here suggested that UMC could be a candidate, since we have detected that the NPCs derived from UMC-iPS cells are less immunogenic. Thus, establishing iPS cell banks derived from UMCs with low immunogenicity could help the development of various clinical therapeutic approaches.

In summary, we mainly investigated the characteristic of immunogenicity in parental somatic cells, their iPS cells and iPS derived NPCs from two cell origins (SF and UMC). The cells derived from SFs and UMCs were compared, where UMC-derived iPS cells and NPCs showed less immunogenicity than the SF-derived cells. It is possible that some other types of somatic cells with low immunogenicity, such as chorionic mesenchymal cells and amnion cells from the extra-embryonic tissues could be used for the production of less immunogenic differentiated cells or tissues that could be used in clinical applications.

## Materials and Methods

All samples were collected following principles approved by the Guangzhou Institutes of Biomedicine and Health Ethical Committee. All volunteers who donated skin or blood samples have provided their written informed consent and the Ethics Committees have approved this consent procedure.

### Cell culture and iPS cell generation

The human adult skin fibroblasts (SFs) from the healthy volunteer were obtained and cultured in DMEM (Hyclone) supplemented with penicillin/streptomycin, L-glutamine and 10% FBS (PAA). Umbilical cord mesenchymal cells (UMCs) derived iPS cells (UMC-iPS cells) used here have been reported to be reprogrammed, having ES-like pluripotent characteristics [15]. SFs derived iPS cells (SF-iPS cells) obtained from South Stem Cell Bank in Guangzhou, were generated by transduction using pMX-based retroviruses by supplement of Vitamin C (Vc) and valproic acid (VPA) as described [15]. HN4-human ES cells (HN4-hES cells) obtained from Hainan Medical College were established as described [61]. HN4-hES cells and two human iPS cells were cultured on feeder layers using KSR medium (DMEM/F12 [Gibco], 20% knockout serum replacement [KSR, Gibco], non-essential amino acids, penicillin/streptomycin, L-glutamine, beta-mercaptoethanol, and bFGF), or on matrigel (BD biosciences) using mTeSR1 medium (Stemcell).

### Neural differentiation *in vitro*

SF-iPS and UMC-iPS cell colonies were detached from feeder layers and grown in suspension for 4 days in KSR medium without bFGF but supplemented with 5 µM dorsomorphin (DM, Sigma) and 5 µM SB431542 (Calbiochem). iPS-derived embryomic bodys (EBs) were treated with neural induction medium containing F12/DMEM, N2 supplement (Gibco), heparin (2 mg/ml), and non-essential amino acids for another 2 days. iPS-derived EBs were then adhered to a matrigel-coated plate. Two weeks later, neural rosettes were blown off and cultured for another 4 days in suspension in N2B27 medium containing DMEM/F12 and Neurobasal medium (1∶1, Gibco), N2 supplement, B27 supplement (Gibco), 20 ng/ml EGF and 10 ng/ml bFGF. Neural sphere (suspension condition of NPCs) were formed and harvested to examine the expression of neuronal markers Nestin and Forse 1. Without EGF or bFGF, NPCs were further cultured for 4 days in N2B27 medium for neuronal induction, or 3 weeks in N2B27 medium supplemented with 0.1% FBS (PAA) for astrocyte differentiation respectively. The results were examined by immunofluorescence microscopy for βIII-tubulin and glial fibrillary acidic protein (GFAP) respectively.

### Isolation of PBMCs and T cells and proliferation assay

Human peripheral blood mononuclear cells (PBMCs) were isolated from blood of the healthy volunteers (n = 20) following density-gradient centrifugation as described everywhere [62]–[65]. Briefly, OptiPrep™ density gradient solutions (Axis-shield) was added in the peripheral blood sample and centrifuged at 900×g for 30 min. PBMCs were collected from the interphase and washed twice with RPMI 1640 medium (Gibco). For direct T cell response assay, CD3+ cells from PBMCs were separated using CD3 MicroBeads (Miltenyi) and a MiniMACS™ Separator with an MS Column. PBMCs or T cells were maintained in RPMI 1640 medium containing 10% FBS (PAA). Cell viability was analyzed by trypan blue exclusion.

The co-culture experiments were carried out with slight modification as described [41], [66]. In detailed, 100 µl/well was set up as a volume system in 96-well plate. 50 µl PBMCs suspension with 4×10^6^ cells/ml was added to 50 µl of each antigen cells containing 200 cells, which was co-cultured for 3 days in 96-well plate. As an antigen, 50 μl suspension of somatic cells (SFs, UMCs), iPS cells (SF-iPS cells, UMC-iPS cells) and NPCs (SF-NPCs, UMC-NPCs) pre-treated with mitomycin C and seeded respectively. PBMCs adding 50 μl medium containing 8 ng/ml phytohemagglutinin (PHA, Sigma) was set up as the positive control group, and adding medium only as the negative control group. For positive and negative groups, the blank group contained only medium without PBMCs in a same final volume (100 μl). For antigen cell stimulated groups, the blank group only contained equal antigen cells as experiment group in 100 μl medium. PBMCs proliferation was evaluated using a cell proliferation BrdU ELISA kit (Roche Diagnostics, Germany) according to the manufacturer's instructions. The reaction was quantified by measuring the optical density at a wavelength of 370 nm and a reference wavelength of 492 nm. The degree of cell proliferation  =  (A370–A492) – (A′_370_–A′_492_) [A: mean absorbance value of experimental group; A′: mean absorbance value of blank group], was calculated for each culture treatment, and final results were summarized with the software Graphpad Prism 5.0.

### Assay of cytotoxic molecules expression in co-culture system

100 µl PBMCs or T cells suspension with 1×10^7^ cells/ml was added to 100 μl mitomycin C treated NPCs suspension each containing 1000 cells in 96-well plate, respectively. For PBMCs, positive and negative control groups were designed as proliferation assay. For T cells, negative control groups only contained the lymphocytes, while cells in positive control group were treated with Dynabeads® Human T-Activator CD3/CD28 (Life technologies) as the manufacturer's instructions. After co-cultured overnight, expression of cytotoxic molecules (perforin and granzyme B) in CD3+CD8− T cells, CD3+CD8+ T cells and CD3−CD56+ NK cells were measured by flow cytometry.

### IFN-γ treatment

IFN-γ treatment was carried out as describe [33]. 25 ng/ml of IFN-γ (PeproTech) was added to NPCs growth media for 48 h, SF-NPCs and UMC-NPCs were then harvested for PCR analysis.

### Real-time quantitative PCR

Total RNA was extracted with Trizol (Invitrogen). 2 µg of RNA was reverse transcribed using RT-PCR kit (Takara) and qPCR was performed using a Thermal Cycler Dice^TM^ Real Time System and SYBR Green Premix EX Taq^TM^ (Takara). β-actin was used for qPCR normalization and all items were measured in triplicate. All primer sequences (5′→3′) are as follows:

HLA-A (NM. 002116.7)

forward “AAAAGGAGGGAGTTACACTCAGG” (1101–1123)

reverse “GCTGTGAGGGACACATCAGAG” (1169–1149)

HLA-B (NM. 005514.6)

forward “TTGTTGCTGGCCTGGCTGTCCTAG” (986–1009)

reverse “CCCTCCTTTTCCACCTGAACTCTT” (1080–1057)

HLA-C (NM. 002117.5)

forward “CTCAGATCACCCAGCGCAAGTT” (556–577)

reverse “AGCGTCTCCTTCCCGTTCTCC” (673–653)

HLA-DPB (NM. 002121.5)

forward “TTCTACCCAGGCAGCATTCAAGTC” (561–584)

reverse “GTCATTTCCAGCATCACCAGGATC” (688–665)

HLA-DQB (NM. 002123.4)

forward “GGAGACCTTCGGGTAGCAACTGT” (113–135)

reverse “GAAGTAGCACATGCCCTTAAACTGG” (229–205)

HLA-DRA (NM. 019111.4)

forward “TGTGGATATGGCAAAGAAGGAGAC” (238–306)

reverse “GGAGGTACATTGGTGATCGGAGTAT” (444–420)

NFκB1 (NM. 002116.7)

forward “AACAGAGAGGATTTCGTTTCCG” (619–640)

reverse “TTTGACCTGAGGGTAAGACTTCT” (722–700)

RelA (NM. 002116.7)

forward “GCATCCACAGTTTCCAGAAC” (466–485)

reverse “CACTGTCACCTGGAAGCAGA” (635–616)

p53 (NM. 000546.5)

forward “CAGCACATGACGGAGGTTGT” (701–720)

reverse “TCATCCAAATACTCCACACGC” (825–805)

Hormad1 (NM. 032132.4)

forward “GCCCAGTTGCAGAGGACTC” (128–146)

reverse “TCTTGTTCCATAAGCGCATTCT” (286–265)

Zg16 (NM. 152338.3)

forward “TCCGGGTCCGAGTCAACA” (229–246)

reverse “TCCTCCAGGTCTCCGTTGC” (328–310)

β-actin (NM. 001101.3)

forward “CCCAGAGCAAGAGAGG” (257–272)

reverse “GTCCAGACGCAGGATG” (621–606).

### Flow cytometry

Somatic cells, iPS cells and iPS derived NPCs were prepared at the concentration of 1.0×10^5^ cells in 100 μl PBS, while PBMCs were prepared at the concentration of 1.0×10^6^ cells in 100 μl PBS. All antibodies including HLA-ABC conjugated to FITC, HLA-DR, DP, DQ conjugated to FITC, Nestin conjugated to PercP-Cy5, CD3 conjugated to PercP, CD8 conjugated to FITC, CD56 conjugated to APC, perforin conjugated to PE and granzyme B conjugated to PE were added and incubated for 30 min at 4°C, after two washes in PBS, cells were acquired and analyzed on a FACS calibur (BD Bioscience). All antibodies above were purchased from BD Biosciences. Diluted (1∶5) mouse anti-human Forse1 IgM (DSHB) was added, and after 30 min incubation at 37°C, cells were washed three times with PBS and incubated with FITC-conjugated goat anti-mouse IgM (Invitrogen) for 30 min at 37°C, and after two washes in PBS, cells were acquired and analyzed.

### Microarray analysis

Total RNA was extracted from SF-NPCs and UMC-NPCs using Trizol (Invitrogen) and the RNeasy kit (Qiagen). Samples were amplified and labeled using a NimbleGen One-Color DNA Labeling Kit. Array hybridization was analyzed with the NimbleGen Hybridization System and followed by washing with the NimbleGen wash buffer kit. The Axon GenePix 4000B microarray scanner was used for array scanning. Genes that have values greater than or equal to lower cut-off: 50.0 in 2 out of two samples were chosen for data analysis. Differentially expressed genes were identified through Fold Change filtering. Pathway Analysis and GO analysis were applied to determine the roles of these differentially expressed genes played in these biological pathways or GO terms. Finally, Hierarchical Clustering was performed to show distinguishable gene expression profiling among samples. The data have been deposited in NCBI's Gene Expression Omnibus and are accessible through GEO Series accession number GSE46647 (http://www.ncbi.nlm.nih.gov/geo/query/acc.cgi?acc=GSE46647).

### Statistical analysis

Results of qPCR were expressed as mean ± SEM in the research and differences among groups were analyzed by one-way analysis of variance (ANOVA) followed by the *t*-tests. Results of PBMCs proliferation and expression of perforin or granzym B were expressed as mean and differences were analyzed by paired-samples *t*-tests. The criterion for statistical significance was set at the 0.05 level. The statistical analysis was performed using SPSS 17.0 programs.

## Supporting Information

Figure S1 **Generation and characterization of hES/iPS cells derived NPCs.** (A) Schematic representation of our NPC differentiation protocol, showing typical EB, neural rosette and neural sphere during differentiation. Scale bar corresponds to 300 μm. (B) Flow cytometry analysis of NPC markers (Nestin and Forse1) in the neural sphere cells derived from HN4-hES, SF-iPS, and UMC-iPS cells. (C) Differentiation potentials of ES/iPS derived NPCs. Top: Phase contrast photographs of single neural rosette differentiated from ES/iPS cells; middle and bottom: immunofluorescence staining for the neuronal marker, βIII-tubulin and astrocyte marker, GFAP respectively with neuron-like and astrocyte-like cells produced from the picked neural rosettes in a further random differentiation experiment. DAPI is shown in blue for all immunofluorescences. Scale bar corresponds to 100 μm.
(PDF)

Figure S2 **Proliferation of PBMCs at the different ratio of responding lymphocytes and stimulator cells.** (A) Proliferation of PBMCs in target cells/PBMCs co-culture system at the ratio of 1∶100 and 1∶1000. (B) Degree value of PBMCs proliferation stimulated by target cells at different ratios. (The raw data used to create Figure S2A with the software Graphpad Prism 5.0.)
(PDF)

Figure S3 **Comparative analysis of HLA-A/B/C and HLA-DPB/DQB/DRA expression in SF-NPC and UMC-NPC after IFN-γ treatment.** The qPCR results were obtained in at least three independent experiments and were expressed as mean ± SEM. A *t*-test was used to compare the various groups and *P*-values less than 0.05 were considered statistically significant. * *P<0.05*.
(PDF)

Table S1 Degree value of PBMCs proliferation stimulated by different cell types derived from SF and UMC. (The raw data used to create Figure 1 with the software Graphpad Prism 5.0.)
(PDF)

Table S2 Percentage of perforin expression in various immune effector cells in PBMCs co-culture system. (The raw data used to create Figure 2B with the software Graphpad Prism 5.0.)
(PDF)

Table S3 Percentage of granzyme B expression in various immune effector cells in PBMCs co-culture system. (The raw data used to create Figure 2C with the software Graphpad Prism 5.0.)
(PDF)

Table S4 Percentage of perforin expression in various immune effector cells in T lymphocytes co-culture system. (The raw data used to create Figure 3B with the software Graphpad Prism 5.0.)
(PDF)

Table S5 Percentage of granzyme B expression in various immune effector cells in T lymphocytes co-culture system. (The raw data used to create Figure 3C with the software Graphpad Prism 5.0.)
(PDF)
