# Supplementary figures and images for: Low Immunogenicity of Neural Progenitor Cells Differentiated from Induced Pluripotent Stem Cells Derived from Less Immunogenic Somatic Cells
Source: PLoS One. 2013 Jul 26;8(7):e69617. doi: 10.1371/journal.pone.0069617 (PMC3724937; doi:10.1371/journal.pone.0069617)

# Figure S1

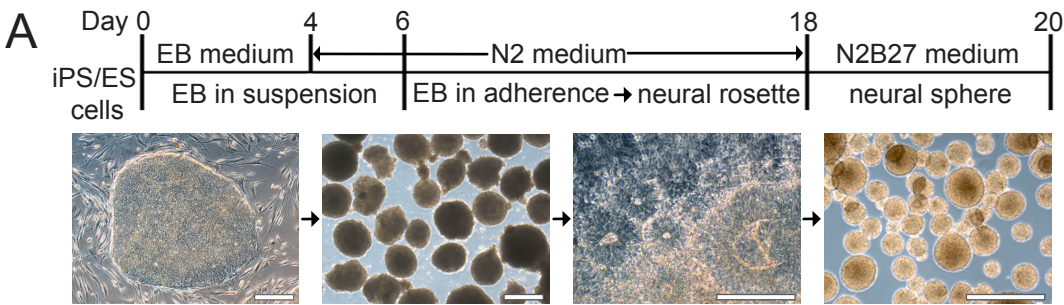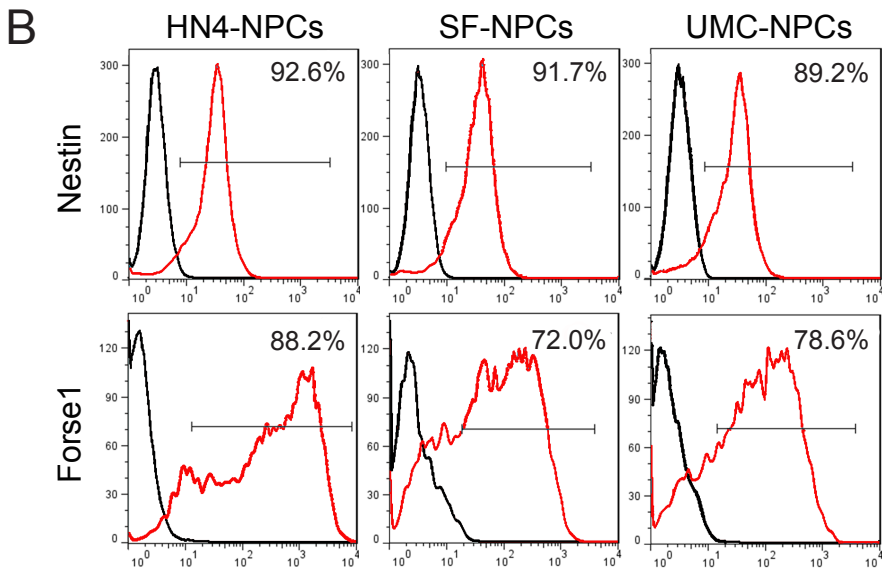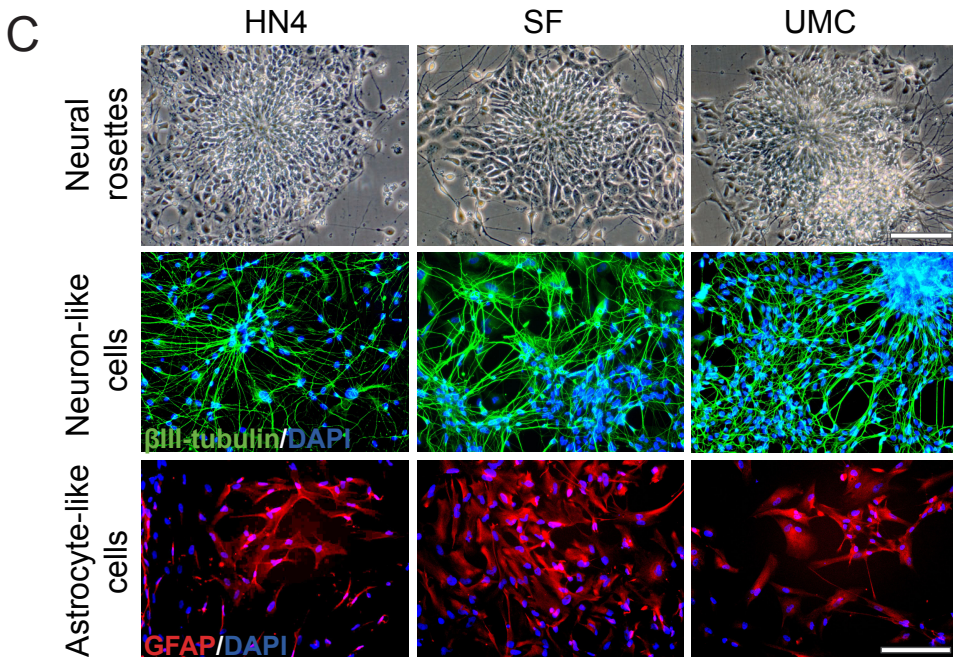

Supplement: Figure S1 — Generation and characterization of hES/iPS cells derived NPCs. (A) Schematic representation of our NPC differentiation protocol, showing typical EB, neural rosette and neural sphere during differentiation. Scale bar corresponds to 300 μm. (B) Flow cytometry analysis of NPC markers (Nestin and Forse1) in the neural sphere cells derived from HN4-hES, SF-iPS, and UMC-iPS cells. (C) Differentiation potentials of ES/iPS derived NPCs. Top: Phase contrast photographs of single neural rosette differentiated from ES/iPS cells; middle and bottom: immunofluorescence staining for the neuronal marker, βIII-tubulin and astrocyte marker, GFAP respectively with neuron-like and astrocyte-like cells produced from the picked neural rosettes in a further random differentiation experiment. DAPI is shown in blue for all immunofluorescences. Scale bar corresponds to 100 μm. (PDF) [file pone.0069617.s001.pdf]

# Figure S2

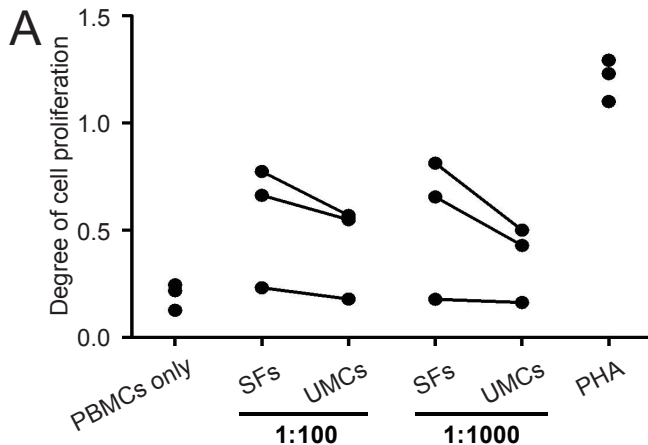

**B**

| No.     | PBMCs only | 1:100 |       | 1:1000 |       | PHA   |
|---------|------------|-------|-------|--------|-------|-------|
|         |            | SFs   | UMCs  | SFs    | UMCs  |       |
| 1       | 0.245      | 0.775 | 0.569 | 0.813  | 0.500 | 1.231 |
| 2       | 0.218      | 0.663 | 0.549 | 0.656  | 0.429 | 1.293 |
| 3       | 0.127      | 0.232 | 0.179 | 0.178  | 0.163 | 1.100 |
| Average | 0.197      | 0.557 | 0.432 | 0.549  | 0.364 | 1.208 |

Supplement: Figure S2 — Proliferation of PBMCs at the different ratio of responding lymphocytes and stimulator cells. (A) Proliferation of PBMCs in target cells/PBMCs co-culture system at the ratio of 1∶100 and 1∶1000. (B) Degree value of PBMCs proliferation stimulated by target cells at different ratios. (The raw data used to create Figure S2A with the software Graphpad Prism 5.0.) (PDF) [file pone.0069617.s002.pdf]

# Figure S3

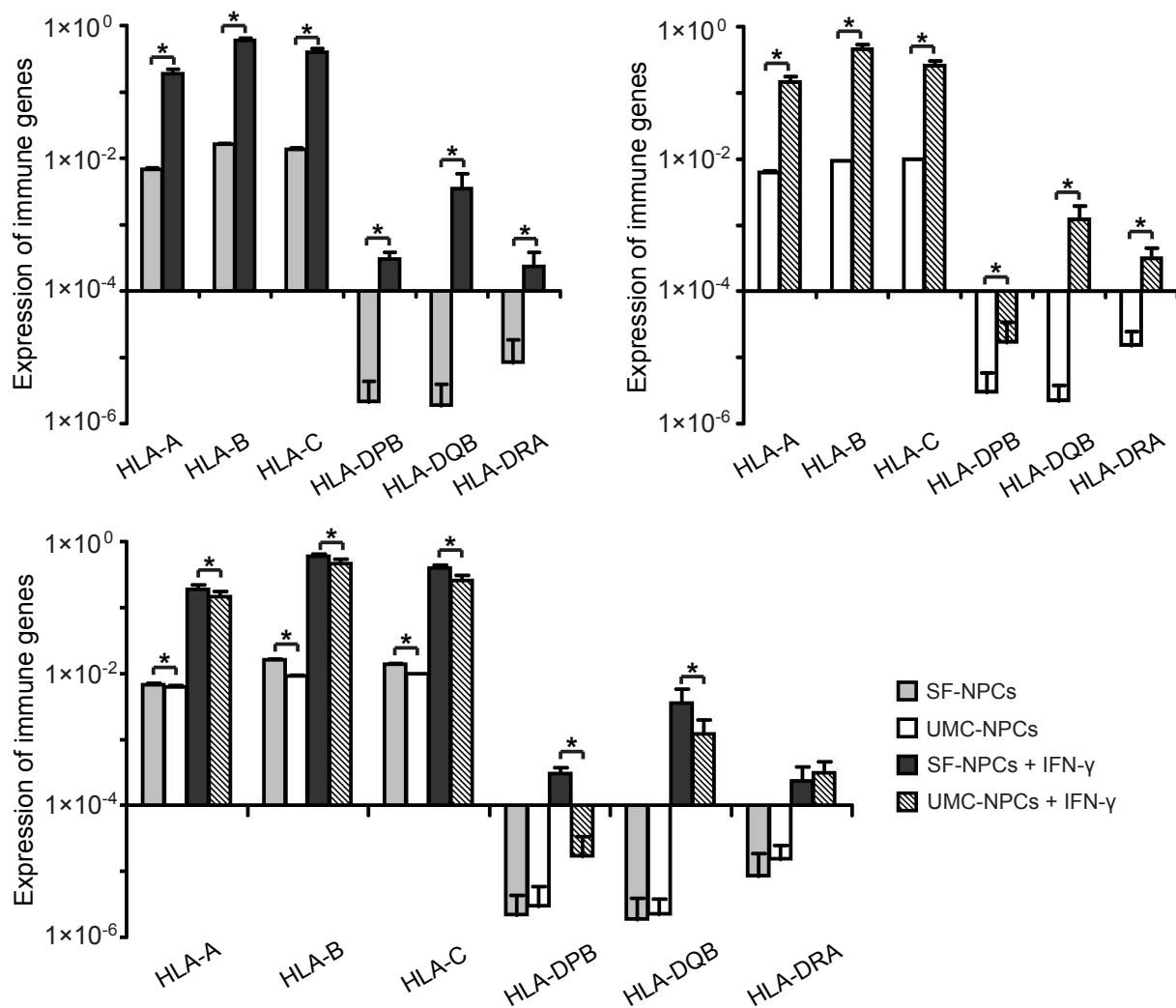

Supplement: Figure S3 — Comparative analysis of HLA-A/B/C and HLA-DPB/DQB/DRA expression in SF-NPC and UMC-NPC after IFN-γ treatment. The qPCR results were obtained in at least three independent experiments and were expressed as mean ± SEM. A t-test was used to compare the various groups and P-values less than 0.05 were considered statistically significant. * P<0.05. (PDF) [file pone.0069617.s003.pdf]
