# Supplementary material for: Low Immunogenicity of Neural Progenitor Cells Differentiated from Induced Pluripotent Stem Cells Derived from Less Immunogenic Somatic Cells
Source: PLoS One. 2013 Jul 26;8(7):e69617. doi: 10.1371/journal.pone.0069617 (PMC3724937; doi:10.1371/journal.pone.0069617)
Supplement: Table S1 — Degree value of PBMCs proliferation stimulated by different cell types derived from SF and UMC. (The raw data used to create Figure 1 with the software Graphpad Prism 5.0.) (PDF) [file pone.0069617.s004.pdf]

Table S1. Degree value of PBMCs proliferation stimulated by different cell types derived from SF and UMC

No.	PBMCs co-cultured with somatic cells				PBMCs co-cultured with iPS cells				PBMCs co-cultured with NPCs			
	PBMCs only	SFs	UMCs	PHA	PBMCs only	SF-iPS cells	UMC-iPS cells	PHA	PBMCs only	SF-NPCs	UMC-NPCs	PHA
1	0.010	0.172	0.140	0.077	0.207	0.207	0.248	0.364	0.175	0.294	0.231	0.544
2	0.129	0.179	0.061	0.154	0.202	0.205	0.173	0.280	0.174	0.271	0.247	0.347
3	0.106	0.172	0.140	0.228	0.426	0.374	0.451	0.732	0.188	0.280	0.208	0.451
4	0.190	0.260	0.240	0.250	0.466	0.483	0.483	0.706	0.074	0.157	0.097	0.164
5	0.214	0.335	0.443	0.388	0.308	0.314	0.422	0.551	0.244	0.297	0.240	0.717
6	0.317	0.463	0.254	0.572	0.195	0.245	0.194	0.719	0.171	0.365	0.257	0.322
7	0.236	0.158	0.121	0.621	0.294	0.329	0.264	0.418	0.190	0.413	0.488	0.524
8	0.048	0.112	0.063	0.157	0.249	0.230	0.194	0.456	0.140	0.277	0.180	0.354
9	0.337	0.337	0.354	0.812	0.340	0.465	0.413	0.687	0.137	0.224	0.164	1.000
10	0.232	0.348	0.328	0.930	0.286	0.350	0.378	0.550	0.135	0.237	0.259	0.934
11	0.292	0.315	0.228	0.828	0.288	0.262	0.328	0.481	0.260	0.476	0.370	0.902
12	0.117	0.324	0.218	0.462	0.106	0.093	0.097	0.327	0.265	0.342	0.294	0.727
13	0.074	0.295	0.197	0.112	0.237	0.276	0.344	0.572	0.356	0.470	0.323	0.836
14	0.321	0.534	0.327	0.412	0.186	0.214	0.232	0.279	0.222	0.234	0.242	0.729
15	0.253	0.217	0.167	0.343	0.319	0.361	0.318	0.436	0.281	0.313	0.233	0.437
16	0.180	0.555	0.520	0.189	0.274	0.189	0.225	0.734	0.214	0.345	0.176	0.604
17	0.134	0.375	0.477	0.419	0.205	0.218	0.183	0.512	0.220	0.207	0.153	0.542
18	0.063	0.470	0.494	0.150	0.261	0.245	0.244	0.447	0.178	0.405	0.370	0.661
19	0.325	0.515	0.510	0.664	0.276	0.292	0.288	0.742	0.265	0.204	0.263	0.538
20	0.172	0.133	0.137	0.168	0.174	0.209	0.168	0.399	0.124	0.133	0.087	0.338
Average	0.187	0.313	0.271	0.397	0.265	0.278	0.282	0.519	0.200	0.297	0.244	0.583

The degree of proliferation = $(A_{370} – A_{492}) – (A'_{370} – A'_{492})$

A: mean absorbance value of experimental group; A': mean absorbance value of blank group
