# Supplementary material for: Low Immunogenicity of Neural Progenitor Cells Differentiated from Induced Pluripotent Stem Cells Derived from Less Immunogenic Somatic Cells
Source: PLoS One. 2013 Jul 26;8(7):e69617. doi: 10.1371/journal.pone.0069617 (PMC3724937; doi:10.1371/journal.pone.0069617)
Supplement: Table S2 — Percentage of perforin expression in various immune effector cells in PBMCs co-culture system. (The raw data used to create Figure 2B with the software Graphpad Prism 5.0.) (PDF) [file pone.0069617.s005.pdf]

Table S2. Percentage of perforin expression in various immune effector cells in PBMCs co-culture system

No.	CD3+CD8- T cells				CD3+CD8+ T cells				CD3-CD56+ NK			
	PBMCs only*	SF-NPCs	UMC-NPCs #	PHA	PBMCs only*	SF-NPCs	UMC-NPCs #	PHA	PBMCs only	SF-NPCs	UMC-NPCs	PHA
1	3.65	5.67	5.45	7.07	3.60	6.23	4.12	7.90	20.70	25.60	19.30	73.20
2	3.91	6.38	6.00	9.06	3.10	4.29	3.37	11.10	4.68	6.43	5.58	9.31
3	3.00	5.87	3.03	6.04	4.60	6.74	5.80	7.30	0.01	4.55	3.65	5.84
4	1.14	2.50	0.91	7.50	3.00	4.70	3.60	9.23	3.57	6.82	3.59	9.50
5	1.01	9.40	1.86	3.75	3.50	10.40	3.90	10.00	0.20	0.89	0.22	0.63
6	1.36	4.04	1.73	4.80	4.90	5.60	4.40	10.00	0.10	0.75	0.46	0.80
7	0.56	1.10	0.91	1.40	0.47	1.37	1.34	1.70	2.68	4.76	6.74	8.22
8	3.67	5.30	4.21	5.61	0.74	4.00	2.99	4.04	2.71	0.58	0.56	7.83
9	4.11	5.00	3.89	7.07	2.18	5.89	4.19	4.68	4.92	12.20	4.18	17.10
10	1.61	1.52	0.90	2.84	1.28	2.78	1.17	2.71	11.40	13.00	18.00	18.30
11	2.00	1.87	1.11	4.01	0.90	1.61	1.20	1.90	5.05	30.70	28.20	28.40
12	7.90	20.40	11.20	22.40	10.00	22.30	14.50	24.10	14.40	15.80	11.40	16.57
13	4.34	4.92	4.48	8.63	3.38	5.39	5.37	8.81	10.30	11.30	9.78	5.64
14	5.42	6.49	7.09	18.00	4.35	6.64	6.01	19.10	16.50	21.60	22.30	24.40
15	8.30	15.10	13.70	15.90	14.40	15.40	14.80	22.35	28.10	38.10	28.30	32.50
16	4.99	5.63	5.75	10.60	4.32	6.25	5.01	12.32	2.96	30.10	19.90	42.40
17	8.30	8.71	8.30	16.20	11.30	14.80	13.70	18.20	17.00	44.10	36.00	52.30
18	0.67	0.78	0.69	1.64	0.57	0.77	0.54	1.29	7.00	9.45	9.16	20.10
19	0.67	1.01	0.69	2.44	0.93	1.51	0.84	2.54	38.20	44.80	47.20	45.20
20	2.04	2.69	2.51	3.69	2.59	3.80	2.84	4.40	32.40	44.40	36.20	45.50
Average	3.43	5.72	4.22	7.93	4.01	6.52	4.98	9.18	11.14	18.30	15.54	23.19

* and #: Equivalent cytotoxic potential based on perforin expression was detected between the above two kinds of T cells in both negative control and UMC-NPC stimulated groups
