# Supplementary material for: Low Immunogenicity of Neural Progenitor Cells Differentiated from Induced Pluripotent Stem Cells Derived from Less Immunogenic Somatic Cells
Source: PLoS One. 2013 Jul 26;8(7):e69617. doi: 10.1371/journal.pone.0069617 (PMC3724937; doi:10.1371/journal.pone.0069617)
Supplement: Table S3 — Percentage of granzyme B expression in various immune effector cells in PBMCs co-culture system. (The raw data used to create Figure 2C with the software Graphpad Prism 5.0.) (PDF) [file pone.0069617.s006.pdf]

Table S3. Percentage of granzyme B expression in various immune effector cells in PBMCs co-culture system

No.	CD3+CD8- T cells				CD3+CD8+ T cells				CD3-CD56+ NK			
	PBMCs only	SF-NPCs	UMC-NPCs	PHA	PBMCs only	SF-NPCs	UMC-NPCs	PHA	PBMCs only	SF-NPCs	UMC-NPCs	PHA
1	15.80	20.00	14.50	44.50	28.30	31.50	29.30	52.30	93.60	96.90	93.30	96.70
2	24.70	27.50	24.50	44.40	18.20	27.30	25.20	53.10	98.30	98.50	98.60	96.90
3	22.20	23.60	23.20	35.50	34.50	41.80	38.20	51.80	69.50	83.80	88.20	97.00
4	24.40	24.10	26.10	26.30	24.50	26.10	24.40	34.00	53.30	79.20	60.00	90.00
5	12.20	14.20	12.90	29.00	10.28	18.20	18.31	41.50	88.10	83.70	84.30	91.70
6	20.20	22.60	19.10	26.40	22.70	23.20	22.10	31.90	79.80	80.60	82.00	88.40
7	12.60	12.30	11.50	20.00	10.20	10.90	10.50	13.30	81.30	87.00	85.20	90.40
8	18.30	19.40	19.80	20.80	16.40	17.80	18.81	20.40	74.70	89.30	87.70	87.80
9	23.60	26.10	25.40	27.20	30.80	31.60	30.20	38.60	83.80	90.80	85.50	99.80
10	30.00	28.20	31.50	37.50	54.70	62.50	57.00	69.90	83.20	87.70	87.50	92.10
11	24.20	25.60	26.50	23.40	35.60	36.80	36.90	36.40	90.10	91.70	90.10	96.40
12	17.90	18.90	19.30	19.90	16.62	18.80	16.00	18.24	85.70	85.10	83.00	90.10
13	26.80	38.40	30.70	38.70	46.80	73.00	65.70	72.90	88.00	97.00	96.70	96.80
14	19.20	20.60	21.60	24.90	25.20	35.60	26.40	24.40	83.40	88.90	86.30	95.50
15	24.70	27.70	24.30	30.40	31.40	33.80	31.00	36.10	60.00	65.70	67.90	77.70
16	31.30	44.30	35.50	45.90	40.20	46.30	40.00	58.00	97.90	98.60	98.20	96.80
17	30.10	30.90	31.80	43.20	43.40	43.70	43.20	52.10	96.70	98.50	96.80	96.70
18	10.20	10.90	10.67	12.70	10.20	12.50	12.20	15.80	98.80	98.70	98.10	97.30
19	16.60	17.20	19.50	20.30	11.60	19.89	17.70	22.60	98.90	99.40	99.50	99.20
20	7.60	9.79	9.39	13.00	14.50	16.50	16.10	18.50	78.10	79.10	77.70	88.70
Average	20.63	23.11	21.89	29.20	26.31	31.39	28.96	38.09	84.16	89.01	87.33	93.30
