# Supplementary material for: Low Immunogenicity of Neural Progenitor Cells Differentiated from Induced Pluripotent Stem Cells Derived from Less Immunogenic Somatic Cells
Source: PLoS One. 2013 Jul 26;8(7):e69617. doi: 10.1371/journal.pone.0069617 (PMC3724937; doi:10.1371/journal.pone.0069617)
Supplement: Table S4 — Percentage of perforin expression in various immune effector cells in T lymphocytes co-culture system. (The raw data used to create Figure 3B with the software Graphpad Prism 5.0.) (PDF) [file pone.0069617.s007.pdf]

Table S4. Percentage of perforin expression in various immune effector cells in T lymphocytes co-culture system

No.	CD3+CD8- T cells				CD3+CD8+ T cells			
	T cells only	SF-NPCs	UMC-NPCs	CD3/CD28	T cells only	SF-NPCs	UMC-NPCs	CD3/CD28
1	0.95	1.66	1.59	5.82	5.04	5.07	3.38	7.20
2	0.51	0.93	0.53	0.55	0.79	2.18	0.96	0.95
3	0.34	0.84	0.47	0.90	0.59	1.70	1.14	1.83
4	0.72	2.20	0.71	5.00	1.34	1.68	1.66	3.30
5	0.76	1.24	0.72	3.06	1.06	1.89	1.26	6.78
6	1.90	2.26	2.38	3.85	1.04	2.18	1.26	3.62
7	1.20	2.38	1.89	2.05	1.27	2.05	1.31	2.49
8	1.79	2.34	2.28	5.45	1.16	1.78	2.22	4.16
9	0.55	0.82	0.76	2.74	0.70	1.90	1.30	3.30
10	0.53	0.96	0.72	1.42	0.98	3.96	0.96	3.95
11	0.58	0.81	0.54	1.05	0.92	1.52	1.31	1.86
12	0.36	1.03	1.10	1.26	0.40	2.50	1.63	2.51
13	1.45	3.09	1.43	2.87	0.74	4.30	3.50	3.05
14	0.31	0.60	0.67	0.99	1.46	3.29	2.11	4.29
15	0.40	0.62	0.44	0.64	1.04	1.46	0.99	1.33
16	0.63	1.51	1.49	1.98	1.01	1.69	1.10	1.71
17	0.80	1.29	1.25	1.33	2.60	2.89	3.80	4.31
18	0.48	0.57	0.48	0.78	0.40	0.76	0.68	1.23
19	2.09	2.19	2.21	4.94	4.75	5.02	5.30	13.50
20	0.77	1.61	0.52	1.73	1.36	2.41	2.19	3.67
Average	0.86	1.45	1.11	2.42	1.43	2.51	1.90	3.75
