# Supplementary material for: Low Immunogenicity of Neural Progenitor Cells Differentiated from Induced Pluripotent Stem Cells Derived from Less Immunogenic Somatic Cells
Source: PLoS One. 2013 Jul 26;8(7):e69617. doi: 10.1371/journal.pone.0069617 (PMC3724937; doi:10.1371/journal.pone.0069617)
Supplement: Table S5 — Percentage of granzyme B expression in various immune effector cells in T lymphocytes co-culture system. (The raw data used to create Figure 3C with the software Graphpad Prism 5.0.) (PDF) [file pone.0069617.s008.pdf]

Table S5. Percentage of granzyme B expression in various immune effector cells in T lymphocytes co-culture system

| No. | CD3+CD8- T cells | | | | CD3+CD8+ T cells | | | |
|---|---|---|---|---|---|---|---|---|
| | T cells only | SF-NPCs | UMC-NPCs | CD3/CD28 | T cells only | SF-NPCs | UMC-NPCs | CD3/CD28 |
| 1 | 1.02 | 5.43 | 5.93 | 6.23 | 1.09 | 13.30 | 13.30 | 14.90 |
| 2 | 3.50 | 4.39 | 3.86 | 4.85 | 3.16 | 4.38 | 3.15 | 6.81 |
| 3 | 2.01 | 2.03 | 2.17 | 23.00 | 4.16 | 4.05 | 4.37 | 5.35 |
| 4 | 0.69 | 1.38 | 0.95 | 1.55 | 0.61 | 1.71 | 1.17 | 2.59 |
| 5 | 2.95 | 3.24 | 3.65 | 3.10 | 6.63 | 7.44 | 5.74 | 7.87 |
| 6 | 6.63 | 7.44 | 5.74 | 7.87 | 15.60 | 17.00 | 17.50 | 26.90 |
| 7 | 4.86 | 5.56 | 5.00 | 6.09 | 12.00 | 11.90 | 11.50 | 14.10 |
| 8 | 5.68 | 7.06 | 5.27 | 6.45 | 9.20 | 12.10 | 11.10 | 22.10 |
| 9 | 2.44 | 3.16 | 3.15 | 8.89 | 4.47 | 6.33 | 6.37 | 8.88 |
| 10 | 4.40 | 13.40 | 11.70 | 15.50 | 12.70 | 18.60 | 16.40 | 27.90 |
| 11 | 3.93 | 5.45 | 4.38 | 8.50 | 16.40 | 19.50 | 18.70 | 26.90 |
| 12 | 8.60 | 9.99 | 9.50 | 13.60 | 12.90 | 14.80 | 14.00 | 26.10 |
| 13 | 5.00 | 5.50 | 5.30 | 10.00 | 13.70 | 13.20 | 13.90 | 19.60 |
| 14 | 4.84 | 6.70 | 5.95 | 9.12 | 8.13 | 12.40 | 8.48 | 22.00 |
| 15 | 5.78 | 6.25 | 5.64 | 8.93 | 10.20 | 14.60 | 12.10 | 23.10 |
| 16 | 10.70 | 12.30 | 11.00 | 17.70 | 8.89 | 10.00 | 8.58 | 16.90 |
| 17 | 3.41 | 3.45 | 3.12 | 4.63 | 8.52 | 9.41 | 9.41 | 10.40 |
| 18 | 11.20 | 11.30 | 11.00 | 14.10 | 9.10 | 10.40 | 9.34 | 11.00 |
| 19 | 0.25 | 4.45 | 1.54 | 2.67 | 0.82 | 6.69 | 1.41 | 4.65 |
| 20 | 6.70 | 6.90 | 8.50 | 7.30 | 7.40 | 10.30 | 8.60 | 12.20 |
| Average | 4.73 | 6.27 | 5.67 | 9.00 | 8.28 | 10.91 | 9.76 | 15.51 |
